# Supplementary material for: The Effects of Indoor Pollutants Exposure on Allergy and Lung Inflammation: An Activation State of Neutrophils and Eosinophils in Sputum
Source: Int J Environ Res Public Health. 2020 Jul 28;17(15):5413. doi: 10.3390/ijerph17155413 (PMC7432088; doi:10.3390/ijerph17155413)
Supplement: Supplementary file 1 [file ijerph-17-05413-s001.pdf]

Supplementary Table S1

Table S1. The results of allergy skin test

Allergens	**Doctor diagnosed asthma** (n=50)		**Healthy** (n=46)	
	Positive n (%)	Negative n (%)	Positive n (%)	Negative n (%)
Dermatophagoides *pteronyssinus,* Derp1	35 (70.0)	15 (30.0)	21 (45.7)	25 (54.3)
Dermatophagoides farina,Derf1	34 (68.0)	16 (32.0)	16 (34.8)	30 (65.2)
Cladosporium herbarium	2 (4.0)	48 (96.0)	0 (0.0)	46 (100.0)
Alternaria alternate	2 (4.0)	48 (96.0)	0 (0.0)	46 (100.0)
Felis domesticus, Cat	15 (30.0)	35 (70.0)	7 (15.2)	39 (84.8)
